# Dynamic domain learning ability enhanced knowledge tracing with stability

**DOI:** 10.1038/s41598-026-53677-z

**Published:** 2026-05-23

**Authors:** Xiuli Diao, Ruiqing Hu, Qingtian Zeng, Zhengguo Song, Hua Duan

**Affiliations:** 1 College of Computer Science and Engineering, Shandong University of Science and Technology, Qingdao, 266590 China; 2 School of Systems Science and Statistics, Beijing Wuzi University, Beijing, 101149 China

**Keywords:** Knowledge tracing, Knowledge forgetting, Knowledge accumulation, Domain learning ability, Transformer, Engineering, Mathematics and computing

## Abstract

Knowledge Tracing (KT) aims to dynamically model a student’s knowledge state to predict future learning performance. However, most existing approaches have two main limitations. On the one hand, they fail to capture the gradual evolution of knowledge over time, overlooking the stable nature of the learning process. As a result, their predictions often show significant temporal fluctuations. On the other hand, they ignore individual differences in students’ abilities across different tasks within the same domain, typically modeling ability as a single, static level, which limits the accuracy of personalized predictions. To address these issues, this research proposes Dynamic Domain Learning Ability Enhanced Knowledge Tracing with Stability (DLAKT). Firstly, unlike previous knowledge tracing methods that primarily rely on knowledge mastery, DLAKT is the first to explicitly incorporate domain learning ability into the KT framework. This design addresses the limitation that knowledge states alone cannot fully capture individual differences. By establishing a clear mapping between skills and multiple ability dimensions, DLAKT constructs an interpretable representation of domain learning ability. The model dynamically adjusts the ability improvement rate according to the student’s knowledge state, response time, and item difficulty, enabling personalized modeling of evolving abilities. Secondly, DLAKT explicitly models knowledge forgetting and accumulation based on memory networks by incorporating multiple learning behavior features, thereby more accurately simulating the learning dynamics. It further introduces a Transformer-based smoothing module to reduce fluctuations in the knowledge state and enhance model stability. Finally, through the joint modeling of knowledge evolution and the dynamic update of domain learning ability, DLAKT achieves more accurate and stable predictions of student performance. Experiments on three real-world educational datasets show that DLAKT consistently outperforms existing mainstream models in prediction accuracy.

## Introduction

With the widespread adoption of large-scale learning platforms, students’ behavioral data during the learning process are systematically recorded, providing new opportunities for a deeper understanding of their knowledge acquisition^1,2^. In this context, Knowledge Tracing (KT), as a key research area in personalized learning^3,4^, aims to dynamically model changes in students’ knowledge states by analyzing their response records and to predict their future learning performance^5^.

**Fig. 1:**
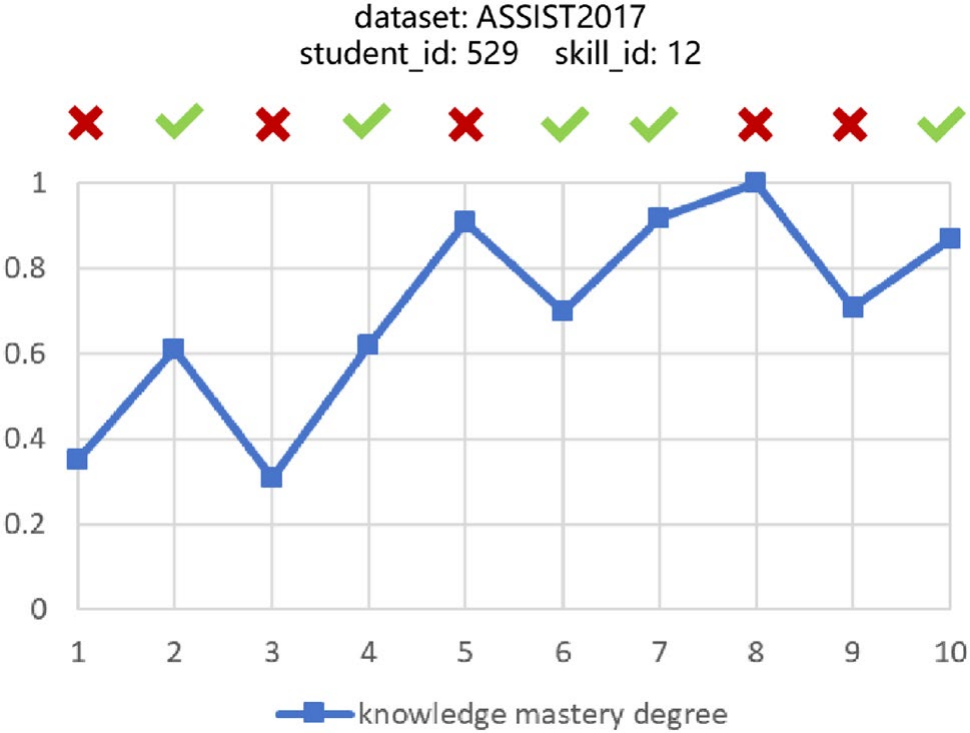
Knowledge state evolution curve of a specific student under DKT.

Early classical models, such as Bayesian Knowledge Tracing (BKT)^6^, assume that knowledge states are discrete variables based on a Hidden Markov Process, making it difficult to capture their continuous evolution over time. With the development of deep learning, Deep Knowledge Tracing (DKT)^7^ uses recurrent neural networks (RNNs) to model response sequences, significantly improving the ability to capture temporal dependencies. Subsequently, a series of DKT extensions are proposed. For example, the Dynamic Key-Value Memory Network (DKVMN)^8^ enhances model interpretability by introducing an external memory mechanism. In existing KT tasks, sparse student interaction data often leads to unstable knowledge state representations, making it difficult to accurately reflect their actual cognitive levels. As shown in Fig. 1, we visualize the knowledge state evolution of a student on a specific skill. It can be observed that the knowledge states predicted by DKT fluctuate frequently across consecutive time steps, lacking a reasonable simulation of the actual learning process. Such dramatic changes fail to reflect the expected stability and gradual progression of knowledge states. This contradicts the fundamental assumption in cognitive psychology that knowledge acquisition occurs through gradual accumulation and smooth changes^9^. It indicates that existing models still have limitations in accurately modeling knowledge states.

Some researches suggest that students’ knowledge acquisition processes are jointly influenced by historical experience and practice content^10^. Therefore, several works attempt to incorporate external information to enhance the stability and consistency of knowledge state representations, such as modeling context through exercise textual content (EKT)^11^ or integrating prior relationships among skills^12–14^. Moreover, the IGKT-GAT variant within the IGKT framework^15^ enhances the model’s sensitivity to the temporal order of learning behaviors by concatenating timestamps and answer correctness as two-dimensional edge features in the student-question graph, demonstrating the potential of temporal features in improving sequential modeling in knowledge tracing. However, these methods still have limitations in fully capturing the richness of students’ cognitive construction. In fact, students’ cognitive construction is a dynamic evolving process, with knowledge mastery levels continuously changing over time^16,17^. Previous research attempts to model this process from perspectives such as review behavior or time decay^18–20^. Furthermore, methods like Learning Process-consistent Knowledge Tracing (LPKT)^21^ and Learning Consistent Representations with Temporal and Causal Enhancement for Knowledge Tracing (TCKT)^22^ reasonably model learning and forgetting behaviors, thereby improving the consistency of learning process representation to some extent. Unlike methods that rely on implicit gating mechanisms to model the evolution of knowledge states, this paper leverages the structural advantages of memory networks and designs an explicit update mechanism that incorporates behavioral features, guided by the cognitive patterns of student learning. This approach enables a more systematic and stable representation of the knowledge acquisition process.

Meanwhile, besides improving prediction performance from the knowledge perspective, modeling student abilities is also an important aspect for enhancing prediction accuracy. Related works such as Deep-IRT^23^, ABKT^24^, DIMKT^25^, IKT^26^, and AA-DKTA^27^ attempt to incorporate factors like student ability and exercise difficulty into model architectures to strengthen predictive accuracy. Although these methods confirm the effectiveness of ability modeling, most focus only on students’ overall ability levels and fail to deeply characterize the multidimensional ability differences within specific domains. In fact, traditional knowledge tracing models usually assume a strong premise. The student’s knowledge mastery solely determines their problem-solving performance. However, educational practice shows that even when students possess the same knowledge points, differences in abilities such as problem comprehension and abstract thinking significantly affect their performance. Therefore, this research introduces the concept of domain learning ability, referring to the multidimensional competency qualities within a discipline that influence student performance, distinguished from the general notion of overall learning ability. For example, key ability dimensions include problem-solving ability, abstract thinking ability, and spatial-geometric ability in mathematics. Moreover, existing research generally lacks dynamic modeling of ability evolution. For instance, OPKT^28^ incorporates knowledge ontology structures to some extent reflecting the impact of different cognitive dimensions on student behavior, but its ontology is fixed before training and lacks a mechanism for modeling ability changes over time. FLAB-IKT^29^ refines ability into multiple dimensions with dynamic updates, however, its ability updating mechanism is relatively simplified, resulting in limited interpretability of this part.

To address the above issues, this paper proposes a knowledge tracing method named Dynamic Domain Learning Ability Enhanced Knowledge Tracing with Stability (DLAKT). Firstly, a domain learning ability modeling module is designed to establish the mapping between skills and domain learning abilities. By incorporating the student’s knowledge state, response time, and exercise difficulty, the module dynamically updates the student’s domain learning abilities from both the student and exercise perspectives. This design allows the model to capture individual differences in domain learning and enhances the interpretability of ability updates. Secondly, the student learning process is divided into knowledge forgetting and knowledge accumulation. Based on the memory network architecture, an update mechanism is designed that integrates three key behavioral features, including the time interval since the last attempt, the interval between attempts on the same knowledge concept, and the total number of attempts. These features enhance the model’s ability to represent knowledge states with greater stability. Specifically, time-related features are primarily used to model the forgetting process, while the number of attempts reflects the accumulation of knowledge. To further suppress temporal noise and improve stability, a Transformer-based smoothing module is introduced. This module incorporates time interval-based positional encoding and leverages the Transformer encoder to extract temporal patterns from the knowledge state sequence. Finally, by integrating the representations of knowledge states and domain learning abilities, the proposed model improves the accuracy of student performance prediction.

The main contributions of this model include:

- We propose a novel Dynamic Domain Learning Ability Enhanced Knowledge Tracing with Stability model. It combines the evolution of students’ domain learning ability with the dynamic changes in their knowledge states to predict future learning performance for the first time.
- A memory network based knowledge state update mechanism is constructed, which explicitly models the processes of knowledge forgetting and accumulation by incorporating key behavioral features such as time intervals and the number of attempts in students’ practice. Meanwhile, a Transformer-based smoothing module is introduced to mitigate noise interference, thereby enabling accurate and stable tracking of students’ knowledge states.
- We propose a dynamic update mechanism for domain learning ability by establishing the mapping between skills and domain learning abilities. By integrating knowledge states and exercise features, we implement incremental dynamic modeling of abilities. This approach not only improves prediction performance but also enhances the interpretability of the ability improvement process.

The remainder of this paper is organized as follows. First, related knowledge tracing methods are reviewed. Next, the knowledge tracing task and relevant concepts are defined. Then, the proposed DLAKT model is introduced in detail. Subsequently, experiments are conducted to validate the effectiveness of the model and compare it with baseline methods. Finally, the paper concludes with a discussion of future research directions.

## Related work

### Traditional knowledge tracing

Traditional knowledge tracing methods played an important role in the early development of this field and mainly fall into two categories including probabilistic models and logical models. Among them, Bayesian Knowledge Tracing (BKT)^6^, as a classical probabilistic model, was widely applied to dynamically model students’ knowledge states. BKT adopted the Hidden Markov Model (HMM) framework, treating a student’s mastery of a specific skill as a latent variable and continuously updating the state based on the student’s responses. Although BKT had a simple structure and effectively described the evolution of students’ knowledge, its binary representation of knowledge was overly idealized and struggled to adapt to the complex and dynamic nature of real-world learning scenarios. In addition, BKT encountered limitations in efficiency and scalability when handling large-scale data.

To enhance modeling capacity, some extended models incorporated additional factors such as problem difficulty (Performance Factors Analysis, PFA^30^), prerequisite relationships among skills (KT-P^31^), and individual student differences (PEBKT^32^), which improved the expressiveness of the models. PFA, as a typical representative of logical models, constructed explicit functional relationships and used students’ past responses to model learning outcomes. However, PFA overlooked potential interactions among factors and failed to fully capture the dynamic changes in student behavior data. Overall, traditional models exhibited certain advantages in interpretability but showed notable limitations in modeling continuous states, capturing temporal dependencies, and generalization performance.

### Deep knowledge tracing

With the development of deep learning techniques^33^, neural network-based knowledge tracing methods gradually became mainstream. Deep Knowledge Tracing (DKT)^7^ was the first to introduce recurrent neural networks (RNN) into KT tasks by feeding students’ response sequences into RNNs or their variant Long Short-Term Memory (LSTM) networks^34^, thereby implicitly modeling students’ knowledge states. Compared with traditional KT models, this approach demonstrated clear advantages in both performance and generalization. However, the knowledge state sequences produced by DKT fluctuated significantly between adjacent time steps, failing to reflect the progressive nature of students’ knowledge acquisition. Subsequent works such as DKT++ and DKT-F attempted to alleviate these fluctuations by introducing regularization or state difference constraints, but overall stability remained unsatisfactory.

To enhance the representation of knowledge structure, the Dynamic Key-Value Memory Network (DKVMN)^8^ introduced a key-value memory mechanism, using a static key matrix to represent concept information and a dynamic value matrix to represent students’ mastery levels over different skills, thereby improving the modeling of knowledge evolution. Building upon this, the Sequential Key-Value Memory Network (SKVMN)^35^ incorporated sequential modeling to capture the influence of temporal information on knowledge updates. DGMN^36^ further integrated knowledge graph structures among problems with memory operations, supporting the modeling of students’ learning and forgetting paths in complex behavioral trajectories. With the advancement of attention mechanisms, SAKT^37^ introduced the Transformer architecture into KT tasks, leveraging self-attention to handle long-range dependencies in students’ response sequences. AKT^38^ proposed a monotonic attention structure and integrated Item Response Theory (IRT)^39^ to enhance the interpretability and prediction reliability of the model. LSEKT^40^ introduced a learning state extraction network to capture students’ current implicit learning states and employed a co-attention mechanism to fuse global and local knowledge mastery information, further improving knowledge state representation and prediction performance. Later models such as DTransformer^41^ and sparseKT^42^ focused on sparsifying the attention mechanism to reduce computational complexity and improve scalability in large-scale applications.

However, despite the significant progress made in structural design and performance improvement, the modeling of stability in students’ knowledge states remained relatively weak. The prediction results often exhibited discontinuities over time, making it difficult to accurately reflect the evolution of students’ cognitive states. To address this issue, some studies began to focus on the inherent patterns of the learning process itself, attempting to explicitly model learning and forgetting mechanisms. This approach aimed to align knowledge state updates with the cognitive characteristics of gradual acquisition and progressive forgetting, thereby improving temporal consistency and representation rationality. For instance, Neural Graph-based Forgetting-aware Knowledge Tracing (NGFKT)^43^ employed heterogeneous graph neural networks combined with a forgetting-aware mechanism to model the graph structure among exercises and the forgetting trajectories in students’ memory, significantly enhancing the model’s robustness to dynamic behaviors. MMAKT^44^ integrated knowledge component relationships, problem difficulty, and forgetting factors using a multimodal attention mechanism to more realistically replicate students’ cognitive learning processes. DIMKT^25^ explicitly incorporated problem difficulty into the modeling process, strengthening the interpretability of answer correctness. Recent studies further explored fine-grained difficulty modeling. For example, Yang et al. proposed the Difficulty-aware Programming Knowledge Tracing (DPKT) model^45^, which leverages large language models to assess both text understanding difficulty and knowledge concept difficulty, demonstrating the potential of difficulty-aware knowledge tracing. LPKT^21^ introduced students’ response times and temporal intervals between exercises to more precisely characterize the temporal dynamics of learning. TCKT^22^ further adopted a causal self-attention mechanism to mitigate bias in exercise data and separately modeled knowledge forgetting and acquisition through forgetting and input gates, improving the consistency of knowledge state representation across dynamic sequences. Nevertheless, most of these methods relied on implicit modeling of learning behaviors through neural networks and lacked explicit constraints on the stability of knowledge state transitions. As a result, prediction outputs still tended to exhibit abrupt changes between consecutive time steps, failing to accurately reflect the progressive nature of students’ cognitive processes.

With further research, models gradually paid more attention to capturing individual differences among students. To enhance personalized prediction, some methods incorporated ability modeling in KT tasks. For example, Deep-IRT^23^ combined student knowledge states obtained from DKVMN with Item Response Theory (IRT), using deep networks to estimate student ability and problem difficulty, thereby improving the interpretability of answer outcomes. ABKT^24^ introduced cognitive ability prior labels within the Bayesian Knowledge Tracing framework to model student ability levels from historical response sequences and capture individual differences. Deep Knowledge Tracing and Dynamic Student Classification (DKT-DSC)^46^ dynamically assessed student ability at each time interval and categorized students accordingly. Students from different categories were then sequentially input into the neural network to improve predictive performance. Although these methods achieved preliminary gains in model effectiveness, they still exhibited notable limitations in ability modeling. (1) Most approaches treated student ability as a static attribute, failing to reflect its dynamic evolution during the learning process, which limited the model’s capacity to capture cognitive development. (2) Most methods employed a single-dimensional overall representation of ability, overlooking the potential variation in ability demands across different knowledge domains and thereby restricting the precision of instructional interventions.

In summary, existing knowledge tracing methods still exhibit limitations in both knowledge state updating and ability modeling. On one hand, the modeling of temporal stability and continuity of knowledge states remains insufficient, making it difficult to simulate the gradual nature of students’ learning processes. On the other hand, most ability modeling approaches treat ability as static and unidimensional, failing to reflect dynamic cognitive differences among students. To address these issues, this study introduces a domain learning ability modeling mechanism that dynamically captures students’ ability levels across multiple cognitive dimensions by incorporating knowledge states, exercise difficulty, and response time. In addition, a Transformer-based smoothing mechanism is introduced to effectively control the rate of change in knowledge states and improve the stability of the model’s representation of student knowledge states.

## Problem formulation

We denote the set of student as $S = \{s_{1},s_{2},...,s_{I}\}$, containing *I* students, and the set of exercise as $Q = \{q_{1},q_{2},...,q_{M}\}$. The set of response results from students *S* answering exercises *Q* is represented as $R = \{r_{1},r_{2},...,r_{t}\}$. Each student’s response outcomes are mutually independent and do not influence one another. The response sequence of student *S* is represented as $X = \{(q_{1},at_{1},r_{1}),it_{1},...,(q_{t},at_{t},r_{t}),it_{t}\}$. Each tuple $(q_{t},at_{t},r_{t})$ constitutes a basic learning unit, where $q_{t}$ denotes the exercise attempted by the student at time step *t*, $at_{t}$ represents the time taken by the student to complete the exercise, and $r_{t}$ is the response outcome. Typically a binary value $r_{t}=1$ indicates a correct response, while $r_{t}=0$ indicates an incorrect one. The term $it_{t}$ denotes the time interval between two consecutive basic learning units.

To enhance personalized prediction, this study introduces domain learning ability modeling.

### Definition 1

**Fig. 2:**
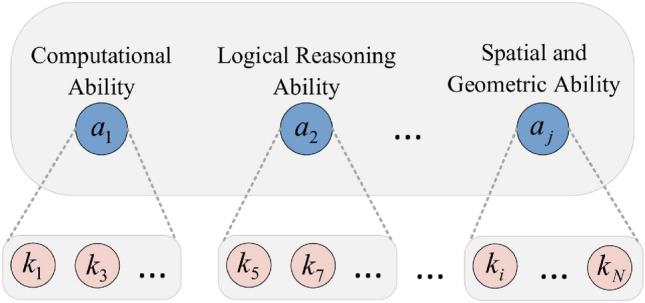
Illustration of the mapping between skills and domain learning abilities. Skills are grouped into higher-level ability categories based on semantic and cognitive similarities, forming a many-to-one mapping.

(*Domain learning ability*) Let the set of skills be $K = \{k_{1},k_{2},...,k_{N}\}$, where *N* is the total number of skills. Based on their names and semantic attributes (e.g., computational, reasoning, spatial), the concepts are grouped into *j* categories corresponding to domain learning ability, forming the ability set $A = \{a_{1},a_{2},...,a_{j}\}$. Each ability represents the core cognitive skills required for a class of related concepts, establishing a many-to-one mapping structure, as illustrated in Fig. 2. Based on this classification, each exercise $q_{m}$ is defined as $q_{m}=(k_{m},A_{m})$, where $k_{m}$ indicates the core knowledge concept involved, and $A_{m}\subseteq A$ represents the set of domain abilities mapped from $k_{m}$. To quantify student performance across different ability dimensions, the domain learning ability at time step *t* is represented by the vector $\boldsymbol{Ability_{t}}= [{Ability_{t}}^{(a_{1})},{Ability_{t}}^{(a_{2})},...,{Ability_{t}}^{(a_{j})}]$, where ${Ability_{t}}^{(a_{j})}$ denotes the student’s ability score in the *j*-th domain at time *t*.

During the learning process, both knowledge state and domain abilities evolve dynamically over time. The knowledge state reflects a student’s mastery of individual skills, while domain abilities reflect performance across broader cognitive dimensions. It is noteworthy that students often exhibit innate differences in specific ability dimensions, and certain abilities (e.g., spatial reasoning, logical inference) are not easily improved through short-term practice. Accurately identifying a student’s weaknesses in specific domain abilities can help tailor more targeted learning strategies. This ability categorization enables the model to distinguish between the varying cognitive demands of different skills and individual differences in the development of domain abilities.

### Definition 2

(*Knowledge tracing*) Based on the modeling objectives, and given a student’s historical interaction sequence *X*, this research aims to trace the evolution of the student’s knowledge state and domain learning abilities and predict the probability that the student correctly attempts a candidate exercise $q_{t+1}$, i.e., $P(r_{t+1}=1|q_{t+1},X)$.

Table 1 presents the formal definitions of the key concepts used in this research.

**Table 1:** Formal definition of important definitions.

Symbol	Text description
*X*	A student’s historical interaction sequence
*A*	The set of domain learning abilities
$\boldsymbol{KF_{t}}$	Knowledge forgetting vector at time step *t*
$\boldsymbol{KA_{t}}$	Knowledge accumulation vector at time step *t*
*λ*	Smoothing coefficient in (0,1), used to weight the original and smoothed knowledge states
*v*	Student ability improvement rate over time
$\boldsymbol{W_{*},b_{*}}$	Weight matrix and bias
$\boldsymbol{{\tilde{M}}_{t+1}^{v}}$	Updated knowledge state matrix, reflecting the effects of knowledge forgetting and accumulation
$\boldsymbol{M_{s}}$	Smoothed knowledge state states
$\boldsymbol{M_{t+1}^{v}}$	Final knowledge state matrix, obtained by weighted fusion of the original and smoothed state matrices
$\boldsymbol{H_{t}}$	Historical state sequence over a time window of length *l* ending at time *t*
$\boldsymbol{H_{t}^{T}}$	Time-encoded enhanced $H_{t}$
$\boldsymbol{Ability_{t}}$	Domain learning ability vector of the student at time step *t*
${\boldsymbol{Ability_{t}}}^{(a_{j})}$	The ability value of a student at time *t* for the *j*-th domain learning ability

## Method

**Fig. 3:**
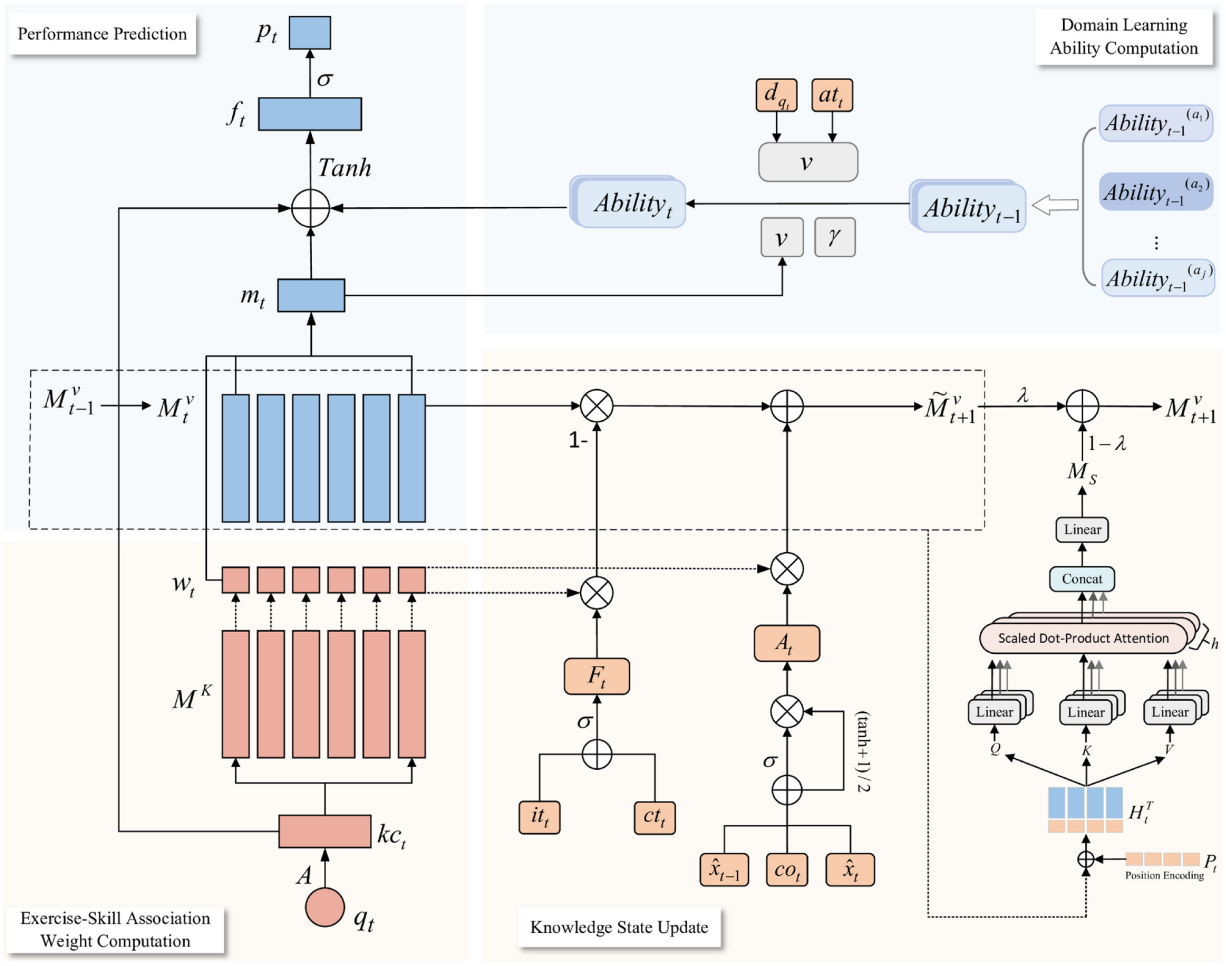
The framework of DLAKT model.

This section presents a novel knowledge tracing model named DLAKT, which aims to improve prediction accuracy by jointly modeling students’ stable knowledge states and dynamic domain learning abilities. The overall model architecture is illustrated in Fig. 3. At each learning step, DLAKT consists of four modules. They are exercise-skill association weight computation, knowledge state update, domain learning ability computation, and response prediction respectively.

### Exercise-skill association weight computation

The exercise-skill association weight computation module captures the relevance between each exercise and all skills. Specifically, the embedding vector of the current exercise $kc_{t}$ queries the key matrix $\boldsymbol{M^{k}}\in {\mathbb {R}}^{I\times d_{k}}$, where each row stores the vector representation of a skill. The resulting correlation weights reflect the degree of association between the exercise and each skill.

Firstly, the exercise label $\boldsymbol{q_{t}}$ is projected via the embedding matrix ${\boldsymbol{A}}\in {\mathbb {R}}^{Q\times d_{k}}$ to obtain the continuous embedding vector $\boldsymbol{kc_{t}}\in {\mathbb {R}}^{d_{k}}$. Then, the inner product between $\boldsymbol{kc_{t}}$ and each memory unit $\boldsymbol{M^{k}}(i)$ in the key memory matrix $\boldsymbol{M^{k}}$ is computed. These values are normalized using the softmax function to generate the correlation weights, as defined in Formula (1).

1 $$\begin{aligned} {\boldsymbol{w_{t}}}(i)=\operatorname {softmax}(\boldsymbol{kc_{t}}^{T}\boldsymbol{M^{k}}(i)) \end{aligned}$$

Where $\sum _{i=1}^{N}\boldsymbol{w_{t}}(i)=1$, indicating the strength of correlation between exercise $\boldsymbol{q_{t}}$ and each skill.

### Knowledge state update

A student’s mastery of each skill is dynamically stored in the value memory matrix $\boldsymbol{M^{v}}\in {\mathbb {R}}^{I\times d_{v}}$. After each exercise response, the knowledge state is updated accordingly. The forgetting mechanism erases outdated knowledge from the value memory matrix, while the accumulation mechanism adds newly acquired knowledge into it. Finally, Transformer-based smoothing is applied to reduce noise and further enhance the stability of the updated knowledge state.

#### Knowledge forgetting

In real-world learning scenarios, a student’s mastery of knowledge tends to decline over time, especially in the absence of timely review. To realistically model the dynamic evolution of student knowledge states, the forgetting mechanism must be capable of capturing complex temporal dependencies. Inspired by Ebbinghaus’ forgetting curve theory^47^, this work incorporates two time-related factors that affect knowledge forgetting: (1) The time interval *it* since the last learning interaction, (2) the time interval *ct* since the last interaction involving the same skill. We represent $\boldsymbol{it_{t},ct_{t}}\in {\mathbb {R}}^{d_{v}}$ as high-dimensional vectors encoding temporal information. Drawing inspiration from the erase and add operations in memory networks, the forgetting process is treated as a selective weakening of previously stored knowledge. A learnable gating structure, conditioned on the combined temporal factors [*it*, *ct*], dynamically adjusts the memory content to reflect the natural decay of a student’s mastery.

A fully connected layer with a *σ* activation function transforms the temporal features into a forgetting vector $\boldsymbol{KF_{t}}\in {\mathbb {R}}^{d_{v}}$, as defined in Formula (2). The value memory matrix is then updated using this forgetting vector, as shown in Formula (3).

2 $$\begin{aligned} & KF_{t}=\sigma (\boldsymbol{W_{f}}^{T}[\boldsymbol{it_{t}}\oplus \boldsymbol{ct_{t}}]+\boldsymbol{b_{f})} \end{aligned}$$

3 $$\begin{aligned} & \boldsymbol{{\tilde{M}}_{t}^{v}}(i)=\boldsymbol{M_{t}^{v}}(i)[1- \boldsymbol{w_{t}}(i)\boldsymbol{KF_{t}}] \end{aligned}$$

Where *⊕* denotes the concatenation operation. $\boldsymbol{W_{f}}\in {\mathbb {R}}^{(2d_{v})\times d_{v}}$ is the weight matrix, and $\boldsymbol{b_{f}}\in {\mathbb {R}}^{d_{v}}$ is the bias vector.

#### Knowledge accumulation

The number of times a student repeatedly engages with the same skill is a key factor in the learning process, reflecting both the compactness and continuity of learning. However, frequency alone is insufficient to capture the complex nature of knowledge accumulation. Existing studies dynamically characterize students’ knowledge state updates through learning gains^21^, but their calculations do not explicitly consider the number of times students review the same knowledge point. To address this, we incorporate the number of times students review the same knowledge point as an additional factor in modeling knowledge accumulation, aiming to more comprehensively reflect the growth of knowledge during learning.

Specifically, we concatenate the exercise $\boldsymbol{q_{t}}\in {\mathbb {R}}^{d_{v}}$, response time $\boldsymbol{at_{t}}\in {\mathbb {R}}^{d_{v}}$, and response $\boldsymbol{r_{t}}\in {\mathbb {R}}^{d_{v}}$. These components are fused through a multilayer perceptron (MLP) to produce the interaction embedding vector $\boldsymbol{{\hat{x}}_{t}}\in {\mathbb {R}}^{d_{v}}$, as defined in Formula (4).

4 $$\begin{aligned} \boldsymbol{{\hat{x}}_{t}}= \boldsymbol{W_{1}^{T}}[ \boldsymbol{q_{t}}\oplus \boldsymbol{at_{t}}\oplus \boldsymbol{r_{t}}]+ \boldsymbol{b_{1}} \end{aligned}$$

Where $\boldsymbol{W_{1}}\in {\mathbb {R}}^{(3d_{v})\times d_{v}}$ is the weight matrix. $\boldsymbol{b_{1}}\in {\mathbb {R}}^{d_{v}}$ is the bias vector.

The modeling of learning gains, or knowledge accumulation, is achieved by concatenating the previous interaction embedding $\boldsymbol{{\hat{x}}_{t-1}}$ with the current interaction embedding $\boldsymbol{{\hat{x}}_{t}}$, which together serve as the basic input element. Additionally, the review frequency embedding $\boldsymbol{co_{t}}\in {\mathbb {R}}^{d_{v}}$ is a key factor in the learning process. Generally, a higher number of reviews on the same skill indicates a more compact and continuous learning trajectory. Based on these factors, the knowledge accumulation $G_t$ is computed as shown in Formula (5).

5 $$\begin{aligned} G_{t}=\tanh ( \boldsymbol{W_{2}}^{T}[ \boldsymbol{{\hat{x}}_{t-1}}\oplus \boldsymbol{co_{t}}\oplus \boldsymbol{{\hat{x}}_{t}}]+\boldsymbol{b_{2}}) \end{aligned}$$

Where $\boldsymbol{W_{2}}\in {\mathbb {R}}^{(3d_{v})\times d_{v}}$ is the weight matrix. $\boldsymbol{b_{2}}\in {\mathbb {R}}^{d_{v}}$ is the bias vector.

Since not all learning gains can be fully converted into knowledge growth, we further introduce a knowledge absorption factor $g_t$ to control the student’s ability to assimilate knowledge, as shown in Formula (6). $KA_{t}$ represents the student’s actual knowledge accumulation at time *t*, as defined in Formula (7).

6 $$\begin{aligned} & g_{t}=\sigma ( \boldsymbol{W_{3}}^{T}[ \boldsymbol{{\hat{x}}_{t-1}}\oplus \boldsymbol{co_{t}}\oplus \boldsymbol{{\hat{x}}_{t}}]+ \boldsymbol{b_{3}}) \end{aligned}$$

7 $$\begin{aligned} & KA_{t}=g_{t}\cdot \left( \frac{G_{t}+1}{2}\right) \end{aligned}$$

Where $\boldsymbol{W_{3}}\in {\mathbb {R}}^{(3d_{v})\times d_{v}}$ is the weight matrix. $\boldsymbol{b_{3}}\in {\mathbb {R}}^{d_{v}}$ is the bias vector.

Since the output range of the tanh function is (-1,1), a linear transformation $(G_{t}+1)/2$ to map $G_t$ from (-1,1) to (0,1). This is consistent with the earlier assumption in the paper and accords with the notion that students gain knowledge consistently through each learning interaction.

Finally, the actual knowledge accumulation $KA_{t}$ is used to update the value memory matrix, resulting in the updated knowledge state matrix $\boldsymbol{{\tilde{M}}_{t+1}^{v}}$, as shown in Formula (8).

8 $$\begin{aligned} \boldsymbol{{\tilde{M}}_{t+1}^{v}}(i)= \boldsymbol{{\tilde{M}}_{t}^{v}}(i)+ \boldsymbol{w_{t}}(i)KA_{t} \end{aligned}$$

Where $\boldsymbol{{\tilde{M}}_{t}^{v}}$ denotes the knowledge state matrix after memory erasure through the knowledge forgetting process, as shown in Formula (3).

#### Transformer-based smoothing

**Fig. 4:**
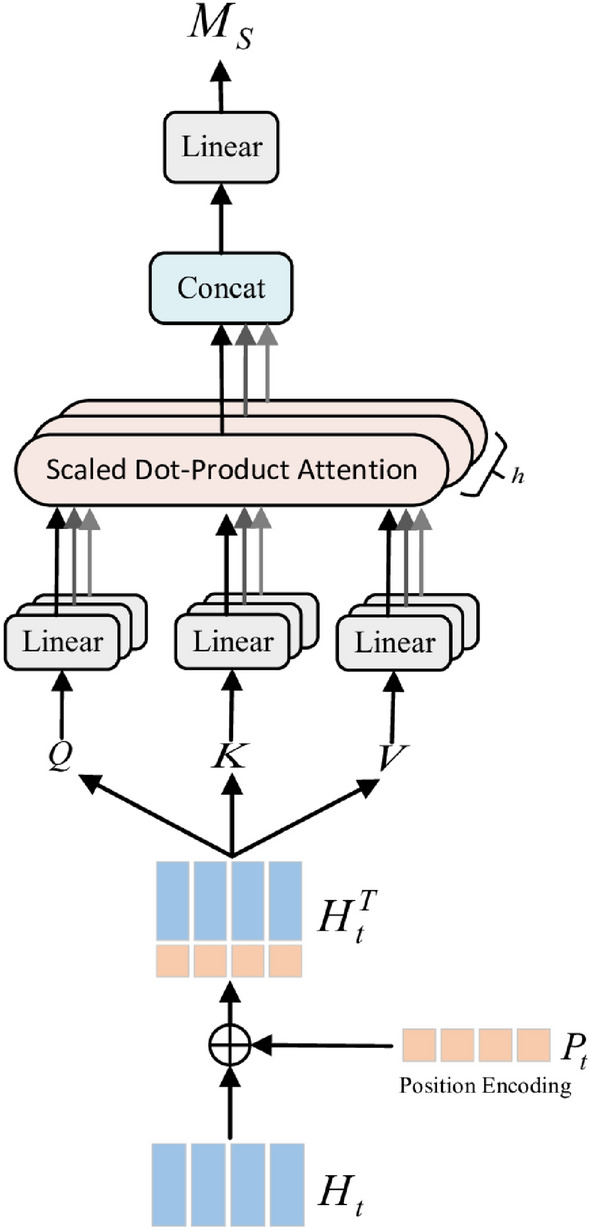
The architecture of transformer-based smoothing module.

Although modeling student behavior can capture the dynamics of knowledge states, knowledge tracing dataset often contains temporal noise. Factors such as fatigue, mood, and randomness may cause brief anomalies in knowledge states, affecting overall trends. If left unaddressed, these anomalies can mislead the model and reduce prediction stability and accuracy. Therefore, after updating knowledge forgetting and accumulation, we introduce a Transformer-based smoothing module as an auxiliary mechanism to reduce noise and enhance the robustness and stability of knowledge state predictions. The structure of the Transformer-based smoothing module is illustrated in Fig. 4.

Knowledge tracing is essentially a sequential modeling task, where the knowledge state at time *t+1* is influenced by prior states at *t-1,t*. Given a time window of length *l*, we construct a historical state sequence for each skill individually, as shown in Formula (9).

9 $$\begin{aligned} \boldsymbol{H_{t}}(i)=\{ \boldsymbol{{\tilde{M}}_{t-l+1}^{v}}(i), \boldsymbol{{\tilde{M}}_{t-l+2}^{v}}(i),\ldots , \boldsymbol{{\tilde{M}}_{t}^{v}}(i)\} \end{aligned}$$

Where $\boldsymbol{{\tilde{M}}_{t}^{v}}(i)\in {\mathbb {R}}^{d_{v}}$ represents the knowledge state vector for skill *i* at time step *t*, after being updated through the processes of knowledge forgetting and accumulation. $\boldsymbol{H_t} (i)\in {\mathbb {R}}^{d_{v}}$ represents the historical state sequence of skill *i* over a time window of length *l* ending at time *t*.

To enhance the model’s perception of temporal dependencies, time interval positional encoding is introduced here. Formulas (10) and (11) define the time interval sequence $T_t$ and its corresponding encoding $P_t$, respectively.

10 $$\begin{aligned} & T_{t}=\{it_{t-l+1},it_{t-l+2},\ldots ,it_{t}\} \end{aligned}$$

11 $$\begin{aligned} & P_t=\tanh ( \boldsymbol{W_{T}}T_{t}+ \boldsymbol{b_T}) \end{aligned}$$

Where $\boldsymbol{W_{T}}\in {\mathbb {R}}^{d_{k}\times d_{k}}$ is the weight matrix. $\boldsymbol{b_{T}}\in {\mathbb {R}}^{d_{k}}$ is the bias vector. The enhanced state sequence $\boldsymbol{H_{t}^{T}}(i)$ is then obtained, as shown in Formula (12).

12 $$\begin{aligned} \boldsymbol{H_{t}^{T}}(i)= \boldsymbol{H_{t}}(i)+P_t \end{aligned}$$

Then, the Transformer encoder architecture^48^ is adopted to extract features from the sequence via multi-head self-attention. As shown in Formula (13). For each attention head, the $\boldsymbol{Q,K,V}$ are obtained by applying different linear transformations to $\boldsymbol{H_{t}^{T}}(i)$.

13 $$\begin{aligned} {\boldsymbol{Q}}= \boldsymbol{H_{t}^{T}}(i) \boldsymbol{W_{Q}}, {\boldsymbol{K}}= \boldsymbol{H_{t}^{T}}(i) \boldsymbol{W_{K}}, {\boldsymbol{V}}= \boldsymbol{H_{t}^{T}}(i) \boldsymbol{W_{V}} \end{aligned}$$

Where $\boldsymbol{W_Q,W_K}\in R^{d\times d_K}$ and $\boldsymbol{W_V}\in R^{d\times d_V}$ are trainable parameters. Then, the attention output is computed based on the scaled dot-product attention mechanism, as defined in Formula (14).

14 $$\begin{aligned} Attention( \boldsymbol{Q,K,V})=\operatorname {softmax}\left( \frac{ \boldsymbol{QK}^{T}}{\sqrt{d_{K}}}\right) {\boldsymbol{V}} \end{aligned}$$

Where $d_K$ denotes the dimension of each attention head, and $\sqrt{d_{K}}$ serves as the scaling factor. The outputs of all attention heads are concatenated and passed through a linear transformation to obtain the smoothed state representation $\boldsymbol{M_{S}}(i)$, as shown in Formula (16), where Formula (15) defines the output of the *h*-th attention head.

15 $$\begin{aligned} & \boldsymbol{M_{S}}^{(h)}(i)=Attention( {\boldsymbol{Q}}_{h}, {\boldsymbol{K}}_{h}, {\boldsymbol{V}}_{h}) \end{aligned}$$

16 $$\begin{aligned} & \boldsymbol{M_{S}}(i)=Concat( \boldsymbol{M_{S}}^{(1)}(i),\ldots , \boldsymbol{M_{S}}^{(H)}(i)) \boldsymbol{W_{O}} \end{aligned}$$

Where $\boldsymbol{M_{S}}^{(h)}(i)$ denotes the output of the *h*-th attention head, and $\boldsymbol{W_O}$ is the projection matrix used to integrate multi-head information. Finally, to enhance the representation capacity and stability, the smoothed features are weighted and fused with the original knowledge state, mimicking a residual connection. The final knowledge state $\boldsymbol{M_{t+1}^{v}}(i)$ is then obtained as shown in Formula (17).

17 $$\begin{aligned} \boldsymbol{M_{t+1}^{v}}(i)=(1-\lambda ) \boldsymbol{{\tilde{M}}_{t+1}^{v}}(i)+\lambda \cdot MeanPool( \boldsymbol{M_{S}}(i)) \end{aligned}$$

Where *λ* *∈*(0,1) is the smoothing coefficient that controls the weighting between the original and smoothed knowledge states. *MeanPool(· )* denotes the mean pooling operation, which adjusts the dimensionality of $\boldsymbol{M_{S}}(i)$ to match $d_v$.

### Domain learning ability computation

**Fig. 5:**
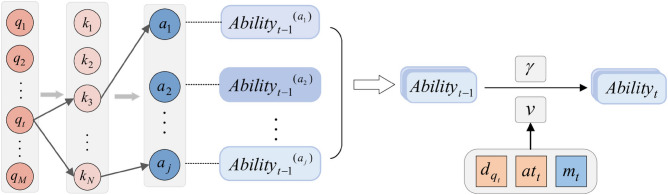
Dynamic model of domain learning ability.

This module characterizes students’ domain learning abilities across multiple cognitive dimensions and dynamically models their evolution throughout the learning process. To support multidimensional ability modeling, a domain expert annotation method is employed. During the annotation, guidelines informed by collective intelligence are followed to associate each exercise with one to three core ability dimensions. Unlike previous approaches that treat student ability as a static variable or model it along a single dimension^23,24,46^, the proposed mechanism classifies abilities into multiple categories and introduces an explicit update method. This method dynamically adjusts each domain ability value based on the student’s knowledge state and exercise features, thereby enhancing the precision and validity of ability modeling while preserving individual differences. The model structure of this module is illustrated in Fig. 5. Specifically, the module analyzes the set of skills associated with the exercise $q_t$ at time step *t*, along with the corresponding domain learning abilities, to selectively update the student’s performance in each ability category, achieving the transition from time *t-1* to *t*.

This study dynamically updates students’ domain learning abilities at time *t*, denoted as $\boldsymbol{Ability_t}$, based on their historical response sequence *X*. The ability update process is described by Formulas (18) and (19).

18 $$\begin{aligned} & { \boldsymbol{Ability}_{1}}^{(a_{j})}= \boldsymbol{Ability}_{0}+\gamma (1-e^{-v}) \end{aligned}$$

19 $$\begin{aligned} & { \boldsymbol{Ability_{t}}}^{(a_{j})}={ \boldsymbol{Ability_{t-1}}}^{(a_{j})}+\gamma (1-e^{-vt}) \end{aligned}$$

The student’s performance across various domain learning abilities is represented as a vector $\boldsymbol{Ability_{t}}\in {\mathbb {R}}^{j}$, where *j* denotes the predefined number of ability categories, such as abstract thinking, modeling and application, spatial geometry in the mathematics domain. This vectorized representation not only enhances the capacity of the model to express ability differences but also supports independent modeling and dynamic updating of each ability, enabling more precise capture of individual student differences and ability evolution trajectories. Where ${ \boldsymbol{Ability_{t}}}^{(a_{j})}$ denotes the student’s proficiency level in domain learning ability $a_j$ at time *t*, $\boldsymbol{Ability}_{0}$ represents the initial domain learning ability before the student starts the response sequence, *γ* is a parameter controlling the maximum improvement range, *e* is the base of the natural logarithm, and *v* is a dynamic parameter controlling the rate of ability improvement.It aims to regulate the improvement speed by jointly considering intrinsic factors (student knowledge state) and extrinsic factors (exercise difficulty and response time). Finally, the definition of the student’s ability improvement rate *v* is provided in Formula (20).

20 $$\begin{aligned} v=\operatorname {Relu}( \boldsymbol{\omega _{1}}\cdot \boldsymbol{m_{t}}+\omega _{2}\cdot d_{q_{t}}-\boldsymbol{\omega _{3}}\cdot \boldsymbol{at_{t}}+b_{v}) \end{aligned}$$

The parameters $\boldsymbol{\omega _{1}},\omega _{2}$, and $\boldsymbol{\omega _{3}}$ are learnable. Specifically, $\boldsymbol{\omega _{1}}$ and $\boldsymbol{\omega _{3}}$ have the same dimensionality as $\boldsymbol{m_t}$ and $\boldsymbol{at}_t$, respectively, and perform inner products with them, while $\omega _{2}$ is a scalar weight and $b_v$ is a bias term. All parameters are optimized via backpropagation using the Adam optimizer to minimize the loss function, and the learned values after convergence are used for model inference. In this paper, exercise difficulty is quantified based on the error rate, defined as the proportion of incorrect responses among all attempts. Specifically, the difficulty $d_{q_t}$ of the exercise $\boldsymbol{q_t}$ attempted by the student at time step *t* is computed as shown in Formula (21).

21 $$\begin{aligned} d_{q_t}=1-\frac{C_{q_{t}}}{N_{q_{t}}} \end{aligned}$$

Where $N_{q_{t}}$ represents the total number of students who answered exercise $\boldsymbol{q_t}$, and $C_{q_{t}}$ denotes the number of students who answered it correctly. The difficulty $d_{q_t}$ ranges from [0, 1], with larger values indicating higher difficulty. Meanwhile, to unify the scale with exercise difficulty, the response time $\boldsymbol{at_t}$ is normalized to [0, 1] using Min-Max normalization.

This structure comprehensively considers multiple factors that influence the improvement of student ability. A higher knowledge state $\boldsymbol{m_t}$ indicates greater potential for improvement, a greater exercise difficulty $d_{q_t}$ leads to higher gains from a single exercise, and a longer response time $\boldsymbol{at_t}$ results in weaker improvement effects. The ReLU activation ensures a non-negative improvement rate, aligning with the educational insight that challenging tasks promote growth. The computation method of the student’s knowledge state $\boldsymbol{m_t}$ is provided in Formula (22).

### Performance prediction

To predict the student’s attempting performance, we first compute the weighted sum of the student’s knowledge state on the skills covered by the current exercise, denoted as $\boldsymbol{m_t}$, as defined in Formula (22).

22 $$\begin{aligned} \boldsymbol{m_t}=\sum _{i=1}^{N} \boldsymbol{w_{t}}(i) \boldsymbol{M_{t}^{v}}(i) \end{aligned}$$

The student’s ability to correctly answer an exercise depends not only on their mastery of the associated skills but also on the characteristics of the exercise itself. To comprehensively model learning behavior, we concatenate the student’s knowledge mastery $\boldsymbol{m_t}$, domain ability vector $\boldsymbol{Ability_{t}}$, and the embedding of the exercise $\boldsymbol{kc_t}$, and feed them into a fully connected neural network to obtain a joint representation of the student’s proficiency. This representation is then passed through another fully connected layer with a *σ* activation function to generate the final prediction. The process is defined in Formulas (23) and (24).

23 $$\begin{aligned} & f_t=\tanh ( \boldsymbol{W_{4}}^{T}[ \boldsymbol{m_{t},kc_{t},Ability_{t}}]+ \boldsymbol{b_{4}}) \end{aligned}$$

24 $$\begin{aligned} & p_t=\sigma ( \boldsymbol{W_{5}}^{T}f_{t}+ \boldsymbol{b_{5}}) \end{aligned}$$

Where $f_t$ is the hidden layer output that integrates three vector representations. $p_t$ represents the predicted probability that the student answers the current exercise correctly. $\boldsymbol{W_{4}, W_{5}}$ are weight matrices, and $\boldsymbol{b_{4}, b_{5}}$ are bias vectors.

### Model optimization

All model parameters, including weight matrices, embedding representations, and ability vectors, are learned by minimizing the cross-entropy loss between the predicted and actual responses. The loss function is defined as follows.

25 $$\begin{aligned} L=-\sum _{t=1}^{T}(r_{t}\log p_{t}+(1-r_{t})\log (1-p_{t})) \end{aligned}$$

Where $r_t$ denotes the ground truth response at time step *t* (1 for correct, 0 for incorrect), and $p_t$ is the predicted probability of a correct response. The logarithm function uses the natural base *e*.

To facilitate a clearer understanding of the DLAKT framework, Algorithm 1 summarizes the main steps of the entire process. The core idea of the algorithm is to model the updates of students’ knowledge states and domain learning abilities during learning, and to use these updates to predict their attempting performance.

**Algorithm 1:**
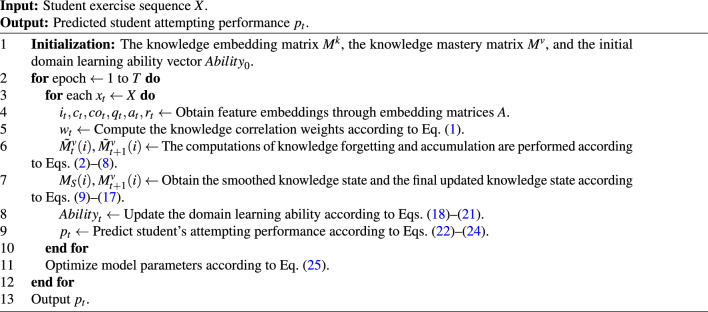
DLAKT algorithm.

## Experiments

This section presents the basic experimental setup, that include datasets, evaluation metrics, comparison methods, and experimental parameters. Then, comparative experiments are conducted on three datasets to verify the advantages of DLAKT. Ablation studies are performed to validate the effectiveness of each module. Finally, knowledge state visualizations are provided to demonstrate the model’s strength in stably tracking knowledge states.

### Datasets

This research employs three real-world online education datasets, namely ASSIST2012, ASSIST2017, and Algebra05. A detailed statistical overview of these datasets is provided in Table 2.

**Table 2:** The statistics of datasets.

Dataset	ASSIST2012	ASSIST2017	Algebra05
# students	3,000	1,709	574
# exercises	34,578	3,162	1,084
# skills	244	102	109
# records	581,040	942,816	606,401
Avg. exercising records per student	193.68	551.68	1056.45

- ASSIST2012 (https://sites.google.com/site/assistmentsdata/home/2012-13-school-data-with-affect): this dataset is collected from the ASSISTments online tutoring system during the 2012-2013 academic year. The original dataset contains responses from 46,674 students to 179,999 problems, resulting in a total of 6,123,270 interaction records. To control the experimental scale, we randomly select a subset of students’ learning trajectories for our experiments.
- ASSIST2017 (https://sites.google.com/view/assistmentsdatamining/dataset): this dataset is collected from the 2017 ASSISTments Data Mining Com-petition and features high data integrity and standardization. Compared to other ASSISTments datasets, it contains longer learning sequences and a greater number of problem-solving records per student.
- Algebra05 (https://kdd.org/kdd-cup/view/kdd-cup-2010-student-performance-evaluation/Data ): the algebra 2005–2006 dataset is released by Carnegie Learning through the PSLC DataShop for the KDD Cup 2010 competition. After preprocessing, the dataset contains 574 students, 109 skills, 1,084 problems, and a total of 606,401 learning interaction records.

To meet the data accuracy requirements of this research, this paper systematically annotates the domain learning abilities associated with each problem $\boldsymbol{q_{t}}$ in the experimental datasets. To ensure the authority and consistency of the annotations, domain experts are invited to manually label each problem based on the cognitive dimensions it assesses. This process establishes a mapping between individual skills and their corresponding domain learning abilities. Taking Algebra05 in the mathematics domain as an example, the covered abilities mainly include computational ability, logical reasoning ability, spatial and geometric ability and function understanding ability. Table 3 presents a selection of domain learning abilities along with their corresponding skills. During the annotation process, we incorporate collective expertise and establish clear annotation guidelines, ensuring that each problem is associated with at least one learning ability and no more than three to maintain both consistency and comprehensive coverage.

**Table 3:** Sample capability annotations for the Algebra05 dataset.

Domain learning abilities	Corresponding skills
Computational ability	Simplifying fractions; integer multiplication; exponentiation; multiplicative inverse; distributive property; factoring quadratics; fraction simplification; solving equations with positive slope involving X
Logical reasoning ability	Addition and subtraction logic; selecting parentheses removal strategies; choosing multiplication/division steps; combining like terms; isolating variables forwardly
Spatial and geometric ability	Plotting coordinate points; labeling axes; selecting vertical reflection of shapes; inputting x-intercepts; adjusting axis ranges; lines included in shading; lines excluded from shading
Function understanding ability	Writing expressions with positive slope; identifying parent function equations; calculating function values Y (in any form); recognizing parent function graphs; writing quadratic function expressions; selecting vertical scaling factors of graphs

### Baseline methods

To evaluate the performance of the DLAKT model, we select the following eleven baseline methods for comparison. A brief description of each is provided below:

- BKT^6^: This model uses binary variables to represent students’ knowledge mastery and employs a Hidden Markov Model to track knowledge state changes and predict problem solving performance.
- DKT^7^: Problems are represented by skills, and an RNN hidden layer is used to trace the evolution of students’ knowledge states, thereby predicting their performance on problems.
- DKVMN^8^: This model employs a key-value memory network to model student learning. The key memory stores skill embeddings, while the value memory stores students’ knowledge states, which are used to predict problem performance.
- DKT+F^19^: An extension of DKT that incorporates features of student forgetting behavior into both model input and performance prediction, enhancing predictive accuracy.
- Deep-IRT^23^: This model combines DKVMN to obtain student knowledge states with Item Response Theory (IRT) to estimate skill difficulty and student ability, enabling prediction of future problem-solving outcomes based on response theory.
- LPKT^21^: It incorporates response time and interval time effects into the learning process and tracks changes in students’ knowledge states.
- LPKT-S^49^: An improvement over LPKT that explicitly distinguishes individual student learning rates.
- GLNC^50^: It introduces a novel global and local neural cognitive model combining cognitive diagnosis and knowledge tracing tasks to improve accuracy and reliability in student performance prediction.
- TCKT^22^: It proposes a knowledge tracing approach enhanced by time and causality factors, introducing a causal self-attention mechanism to reduce prediction errors caused by dataset bias. It models knowledge forgetting and acquisition via a forget gate and input gate, respectively, improving consistency in knowledge state representation.
- LefoKT^51^: This method overcomes the limitations of fixed-window prediction by simulating diverse forgetting behaviors in long-sequence interactions, thereby enhancing its ability to perform long-range inference on the dynamic evolution of knowledge states.
- CFGKT^52^: It presents a dual-granularity state-aware architecture, with a coarse-grained module diagnosing skill-specific proficiency and a fine-grained module quantifying accumulated experience. A state fusion attention mechanism dynamically integrates both states for deep knowledge tracing and interpretable prediction.
- MBFKT^53^: By integrating educational psychology theories and student behavior features, this model precisely represents the learning process as memory decay, memory strengthening, and memory updating phases, significantly improving the accuracy and interpretability of knowledge state tracing.

### Experimental settings

To ensure the reliability and comparability of experimental results, this study adopts standardized procedures for data preprocessing and model training. During the data preprocessing stage, raw data are cleaned by removing interaction records that lack key fields such as exercise IDs or knowledge concept tags. Student response sequences are then sorted by timestamps to maintain temporal consistency in the learning process. For data partitioning, all student interaction sequences are divided into training and test sets at an 8:2 ratio. All models are trained on the training set and directly evaluated on the test set. To ensure fair comparison among different methods, all baseline models are carefully fine-tuned to achieve their best performance on the same dataset.

A consistent set of hyperparameters is used throughout the experiments: the batch size is set to 32, the Adam optimizer is applied with a fixed learning rate of 0.001, and dropout regularization is introduced to prevent overfitting. All models are implemented using the PyTorch framework and trained in a Python environment on a Linux server equipped with an Intel^®^ Core™ i5-12500H @ 2.50 GHz and an NVIDIA Tesla P100 GPU with 16GB of memory. This standardized experimental setup ensures consistency and reproducibility, providing a reliable basis for comparing the performance of different methods.

### Evaluation metrics

In this study, to assess the predictive capability of the models, we adopt two commonly used evaluation metrics: the Area Under the Curve (AUC)^54^ and Accuracy (ACC)^55^. AUC measures the ability of the model to distinguish between positive and negative samples. A value closer to 1 indicates better performance, while a value of 0.5 suggests no better than random guessing. Accuracy reflects the proportion of correctly predicted samples among all predictions and serves as a key indicator of overall prediction precision.

### Experimental results

In this subsection, we discuss the advantages of the proposed DLAKT model over baseline methods. The effectiveness of the model is validated through both comparative and ablation experiments. In addition, visualization experiments are conducted to demonstrate the contribution of the model to the stability of knowledge state.

#### Embedding dimension

**Fig. 6:**
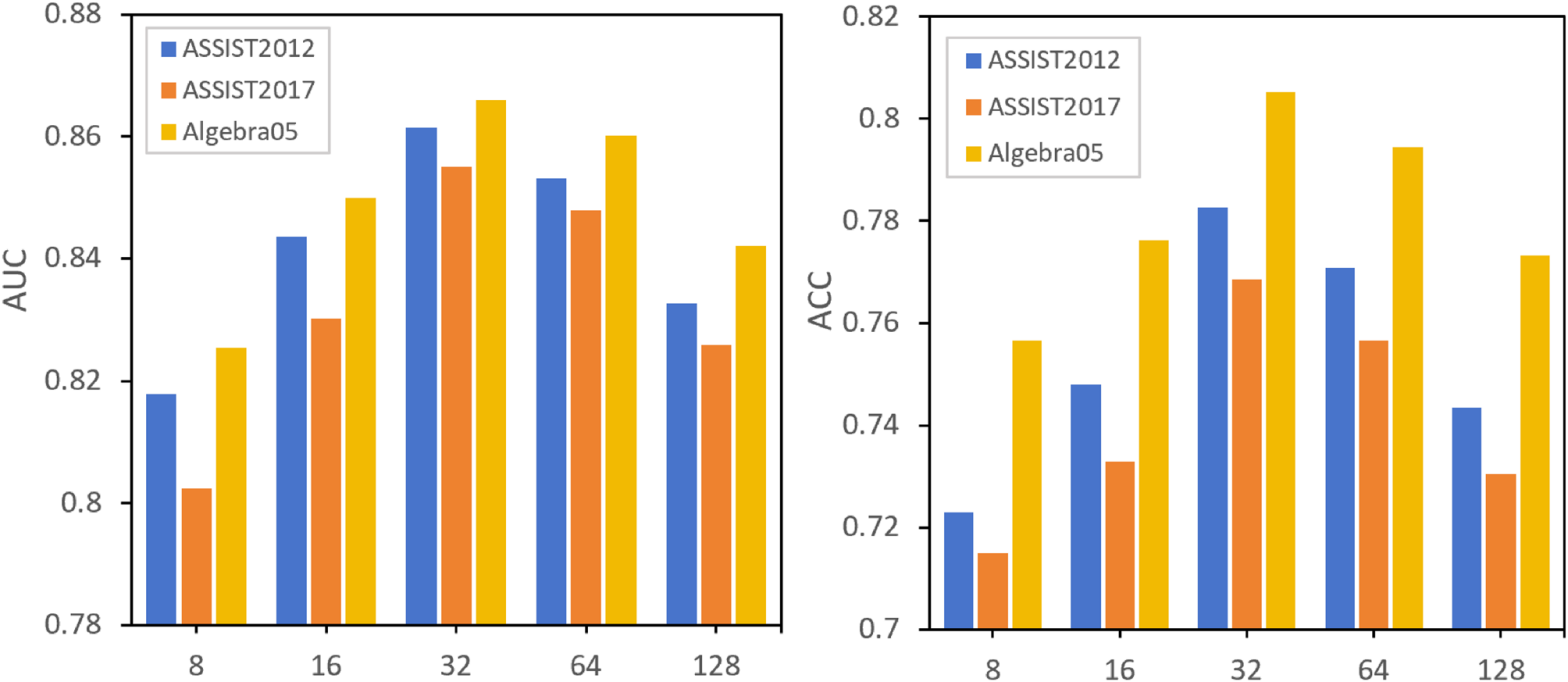
Performance of DLAKT under different embedding dimensions.

To reduce the number of parameters, the dimensionality of the skill embedding vector $d_k$ and the student knowledge mastery embedding vector $d_v$ is constrained to be equal, i.e.,$d_{k}=d_{v}$. Figure 6 illustrates the performance of the DLAKT model across three datasets as the embedding dimension varies from 8 to 128. The predictive performance of the model is evaluated using two metrics, AUC and ACC.

The experimental results show that the model achieves the best overall performance at an embedding dimension of 32. As the embedding dimension increases from 8 to 32, the performance of DLAKT improves significantly, indicating that higher-dimensional representations offer stronger expressive capacity, enabling the capture of more latent skill features and student knowledge states. However, further increasing the dimension to 64 or 128 leads to a noticeable performance drop across all three datasets, suggesting that excessively high dimensions may introduce redundant information and cause overfitting, thus compromising the generalization ability of the model. Moreover, the performance trends observed across the three datasets are consistent, confirming that a moderate embedding dimension helps strike a balance between representation power and generalization across various educational settings. Based on these findings, an embedding dimension of 32 is adopted as the default setting in the subsequent experiments.

#### Impact of different *λ* values

**Fig. 7:**
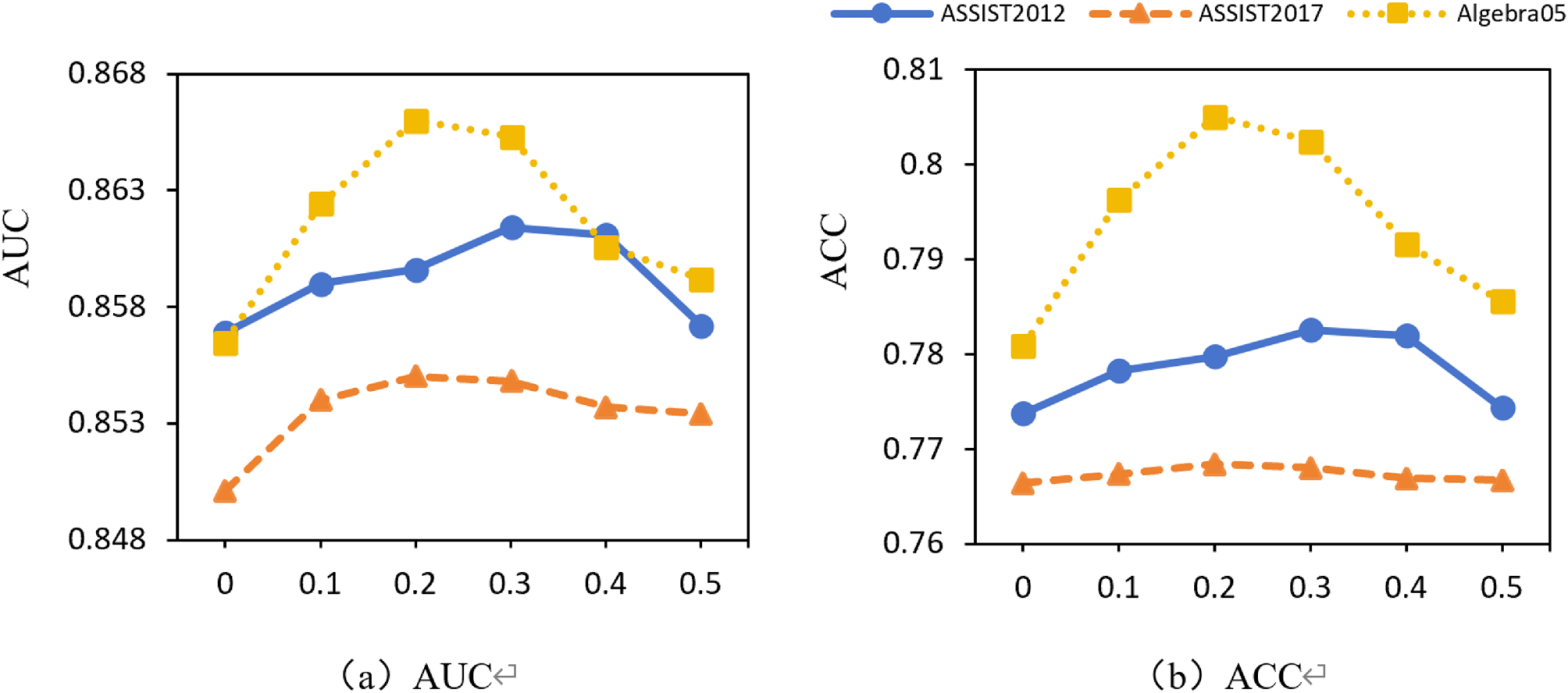
Impact of different *λ* values on the AUC and ACC of DLAKT.

In this paper, the Transformer smoothing module plays an auxiliary role in stabilizing knowledge states. Accordingly, the parameter *λ* is set within the range [0–0.5]. Figure 7 illustrates the effect of different *λ* values on the performance of the DLAKT model in terms of AUC (Fig. 7-a) and ACC (Fig. 7-b) across the three datasets.

The experimental results indicate that the best performance is achieved when *λ*=0.3 on the ASSIST2012 dataset, and *λ*=0.2 on both the ASSIST2017 and Algebra05 datasets. However, when *λ* increases further to 0.5, performance degrades across all datasets. These findings suggest that a moderate degree of Transformer-based smoothing can effectively stabilize the knowledge state. In contrast, excessive smoothing may impair the ability of the model to capture the dynamic evolution of knowledge, thus leading to reduced performance.

#### Performance comparison

To verify the effectiveness of the proposed model, comparative experiments are conducted on three real datasets against eleven representative knowledge tracing models. The results are shown in Table 4.

**Table 4:** Comparative results of different models on three datasets.

Models	ASSIST2012		ASSIST2017		Algebra05	
	AUC	ACC	AUC	ACC	AUC	ACC
BKT	0.6732	0.6310	0.5620	0.5550	0.6370	0.6112
DKT	0.6939	0.7264	0.7247	0.6212	0.7860	0.7723
DKVMN	0.6944	0.7125	0.6620	0.6717	0.7998	0.7841
DKT+F	0.7220	0.7416	0.7258	0.6108	0.7962	0.7835
Deep-IRT	0.7789	0.7432	0.7803	0.7621	0.8013	0.7867
LPKT	0.7704	0.7538	0.7978	0.7391	0.8003	0.7836
LPKT-S	0.7801	0.7562	0.7989	0.7420	0.8180	0.7865
GLNC	0.7869	0.7208	0.7717	0.7034	0.7984	0.7301
TCKT	0.7721	0.7689	0.7961	0.7330	0.7824	0.7645
LefoKT	0.8110	0.7677	0.8356	0.7758	0.8134	0.7715
CFGKT	0.7389	0.7673	0.7756	0.7186	0.7638	0.7974
MBFKT	0.8412	0.7937	0.8329	0.7678	0.8563	0.7982
DLAKT (ours)	0.8614	0.7826	0.8550	0.7685	0.8660	0.8051

As shown in Table 4, DLAKT achieves the highest AUC scores across all datasets, and consistently outperforms other methods in terms of ACC, demonstrating strong predictive performance and generalization ability. Traditional models such as BKT perform poorly due to their simplified assumptions, which limit their capacity to capture the dynamic evolution of students’ knowledge states. RNN-based models such as DKT and its variant DKT+F offer improvements in temporal modeling, but fall short in characterizing complex learning behaviors and often suffer from unstable knowledge state predictions. DKVMN enhances the representation of knowledge states through an external memory mechanism. While Deep-IRT further incorporates item response theory to better capture student ability and item difficulty.

Furthermore, compared with methods such as LPKT, LPKT-S, and TCKT that implicitly model the student learning process, our model explicitly represents knowledge state transitions by leveraging the structural advantages of memory networks. This design enhances the temporal responsiveness and stability of the knowledge state updates, leading to superior predictive performance. In addition, GLNC enhances model expressiveness by introducing a global-local neural structure that integrates cognitive diagnosis into the knowledge tracing task. However, its architecture is relatively complex and highly dependent on data. CFGKT adopts a coarse-to-fine state fusion strategy to improve interpretability, but it heavily relies on the fusion mechanism, making it susceptible to instability due to imbalanced module weights. MBFKT incorporates theories from educational psychology to model students’ memory dynamics and shows certain advantages in terms of ACC. Nonetheless, it lacks explicit modeling of student abilities, leaving room for improvement in predictive accuracy. This further highlights the effectiveness of our domain learning ability module in enhancing personalized prediction performance.

Overall, DLAKT strikes a better balance among accuracy, consistency, and generalization, exhibiting superior knowledge tracing capability. Additionally, we observe that DKVMN underperforms DKT on ACC and AUC in ASSIST2012 and ASSIST2017. This may be due to the key matrix construction of DKVMN, which overlooks correlations among skills^22^, leading to slightly lower performance on certain metrics.

#### Ablation study

To further verify the effectiveness of each module in the proposed model, this section presents a series of ablation experiments. Table 5 shows the core component configurations of each variant. These variants are evaluated on the three datasets, and the results are presented in Table 6.

- DLAKT-NoA: This variant removes the knowledge accumulation process.
- DLAKT-NoF: This variant removes the knowledge forgetting process.
- DLAKT-NoN: This variant simultaneously removes both the forgetting and accumulation modules.
- DLAKT-NoDA: This variant removes the domain learning ability module.
- DLAKT-NoT: This variant removes the Transformer-based smoothing module.

**Table 5:** Comparison of module composition in variant models.

Model	Knowledge accumulation	Knowledge forgetting	Transformer-based smoothing	Domain learning ability
DLAKT-NoA		*\checkmark*	*\checkmark*	*\checkmark*
DLAKT-NoF	*\checkmark*		*\checkmark*	*\checkmark*
DLAKT-NoN			*\checkmark*	*\checkmark*
DLAKT-NoDA	*\checkmark*	*\checkmark*	*\checkmark*	
DLAKT-NoT	*\checkmark*	*\checkmark*		*\checkmark*
DLAKT	*\checkmark*	*\checkmark*	*\checkmark*	*\checkmark*

The ablation results demonstrate that each component of the proposed model contributes positively to overall performance. Among the variants, DLAKT-NoDA exhibits the most significant performance drop, with an average AUC decrease of over 3 percentage points and a notable decline in ACC. This highlights the critical role of domain learning ability in enhancing the model’s generalization and capturing long-term learning behaviors. Both DLAKT-NoA and DLAKT-NoF lead to noticeable performance degradation. This confirms the importance of modeling knowledge accumulation and forgetting for capturing students’ continuous learning progress and knowledge state fluctuations. DLAKT-NoN shows an even larger performance drop than removing either module alone. This indicates that accumulation and forgetting are complementary and both essential for dynamic knowledge modeling. DLAKT-NoT results in a slight performance decrease, but overall effectiveness remains strong, reflecting the auxiliary role of temporal smoothing in enhancing the stability of knowledge states. Overall, DLAKT consistently achieves the best results across all datasets. This indicates that the collaborative design of domain learning ability and dynamic knowledge modeling mechanisms is the key factor in improving prediction accuracy.

**Table 6:** The AUC and ACC results of ablation experiment.

Models	ASSIST2012		ASSIST2017		Algebra05	
	AUC	ACC	AUC	ACC	AUC	ACC
DLAKT-NoA	0.8370	0.7462	0.8293	0.7391	0.8437	0.7602
DLAKT-NoF	0.8397	0.7466	0.8356	0.7425	0.8523	0.7668
DLAKT-NoN	0.8306	0.7423	0.8217	0.7419	0.8410	0.7543
DLAKT-NoDA	0.8211	0.7401	0.8130	0.7232	0.8246	0.7427
DLAKT-NoT	0.8569	0.7738	0.8501	0.7664	0.8565	0.7809
DLAKT	0.8614	0.7826	0.8550	0.7685	0.8660	0.8051

#### Generalization validation of the domain learning ability

To systematically evaluate the compatibility and generalization of the domain learning ability module, we integrate it into three representative knowledge tracing models, including DKT, DKVMN, and LPKT, for comparative experiments. The domain learning ability module is integrated into each of them for comparative experiments. These models represent three distinct architectural paradigms, which are sequential prediction models, memory augmented networks and learning process modeling methods, respectively. This selection enables a comprehensive assessment of the module’s adaptability across different modeling frameworks. We conduct performance evaluations on all three datasets, and the results are presented in Table 7. The results indicate that, in most cases, incorporating the domain learning ability module significantly improves the predictive performance of the baseline models across all datasets.

**Table 7:** The AUC and ACC results of ablation experiment.

Models	ASSIST2012		ASSIST2017		Algebra05	
	AUC	ACC	AUC	ACC	AUC	ACC
DKT	0.6939	0.7264	0.7247	0.6212	0.7860	0.7723
DKT+DA	0.7210	0.7396	0.7564	0.6409	0.8096	0.7831
DKVMN	0.6944	0.7125	0.6620	0.6717	0.7998	0.7841
DKVMN+DA	0.7321	0.7465	0.7031	0.6956	0.8112	0.7894
LPKT	0.7704	0.7538	0.7978	0.7391	0.8003	0.7836
LPKT+DA	0.7963	0.7688	0.8240	0.7576	0.8202	0.7993

#### 3D visualization of domain ability improvement rate

**Fig. 8:**
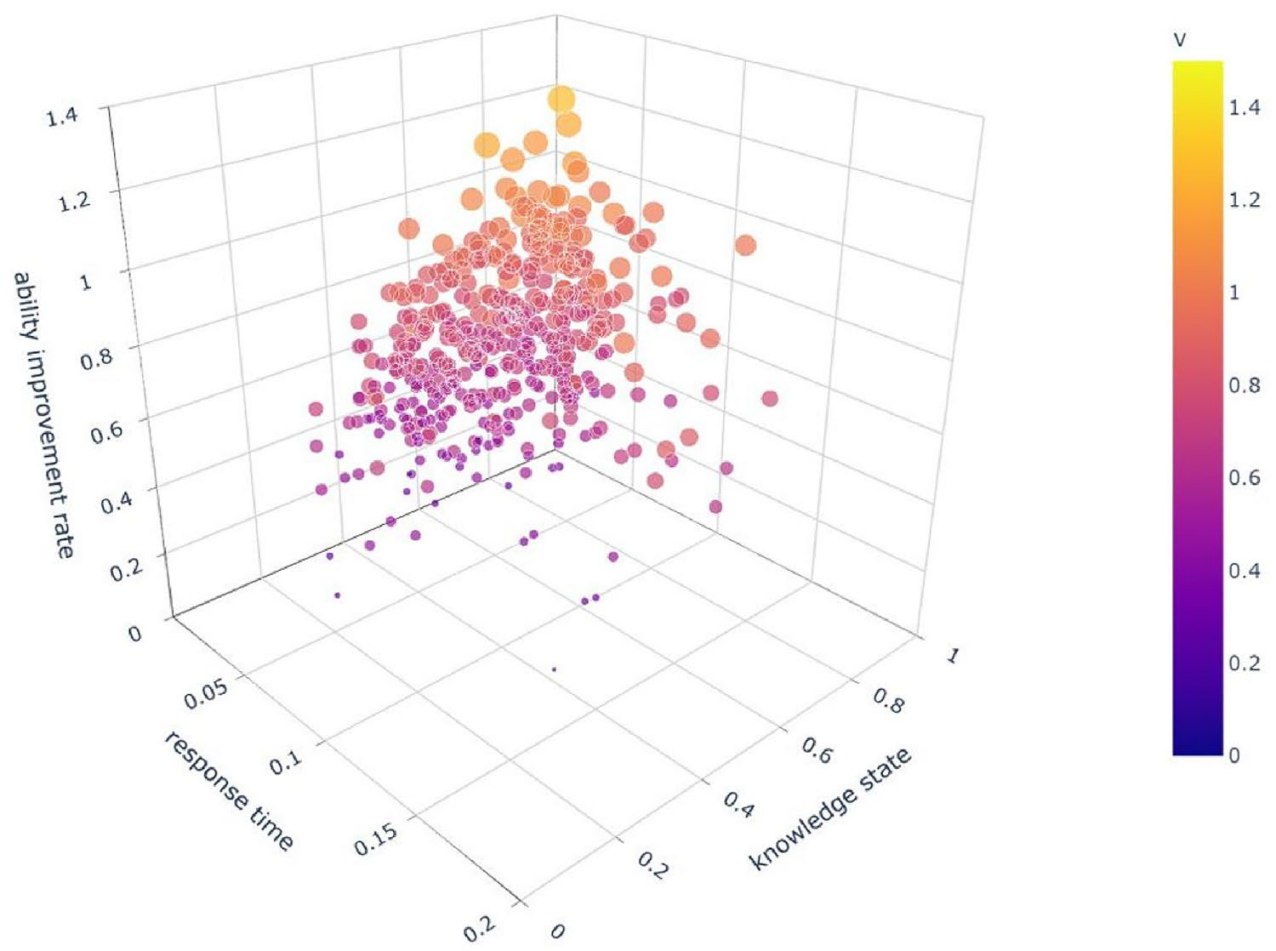
3D visualization of domain ability improvement rate *v*.

To further analyze the relationship between students’ knowledge states, response time, exercise difficulty, and ability enhancement during the learning process, we present a 3D scatter plot of the ability improvement rate *v* constructed on the ASSIST2017 dataset, as shown in Fig. 8. In the figure, the X-axis represents response time, the Y-axis indicates the knowledge state, and the Z-axis indicates the domain ability improvement rate *v*. The size of each point reflects the relative difficulty of the exercise, while color is used to emphasize the variation in the value of *v*.

The visualization results indicate that the rate of ability improvement tends to be higher when the knowledge state is relatively strong and the response time is short. This suggests that students are more likely to achieve effective ability gains when they have a solid understanding of the material and complete exercises fluently, which aligns with the principle of timely feedback in learning. In addition, larger data points, representing more difficult exercises, are often associated with higher rates of ability improvement. This implies that challenging exercises can promote more significant enhancements in student abilities to some extent. The visualization effectively demonstrates the proposed capability in capturing the ability improvement process and provides an intuitive explanation for the proposed ability mechanism. Through three dimensional visualization, the proposed model effectiveness in capturing the interactions among knowledge state, response time, exercise difficulty, and ability dynamics becomes more evident.

#### Knowledge state visualization

**Fig. 9:**
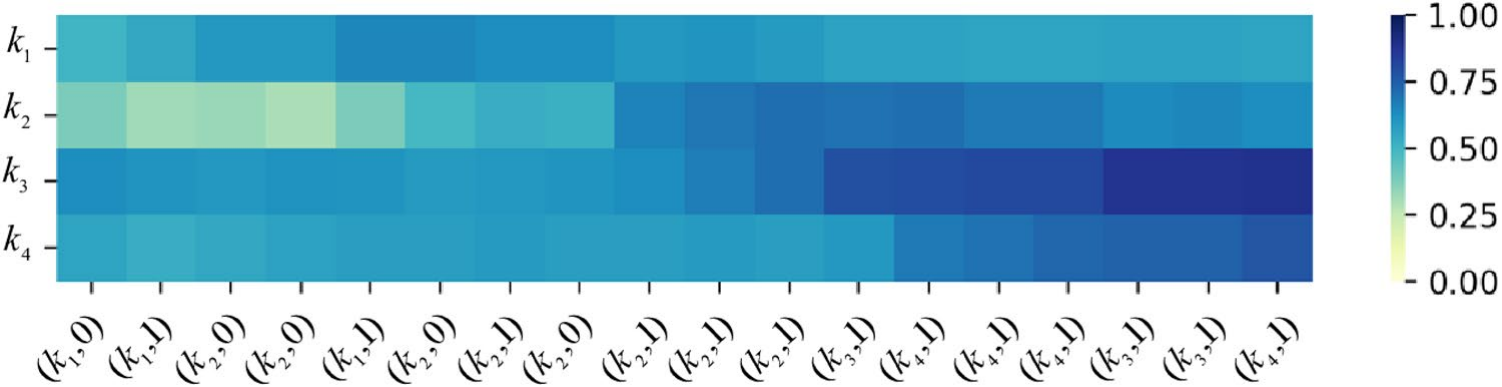
Knowledge state visualization.

To further verify the ability of the proposed model to accurately capture the evolution of students’ knowledge states, we conduct a visualization analysis on the ASSIST2017 dataset. We select student interaction data over 18 consecutive time steps and focus on four representative skills $k_1$(Proportion), $k_2$(Application: Simple Multiplication), $k_3$(Divisibility) and $k_4$(Mode). These skills span both fundamental operations and statistical reasoning, and represent different dimensions of domain learning ability, thereby ensuring broad representativeness. In Fig. 9, the horizontal axis represents each time step as a tuple $(k_i,r_t)$, where $k_i$ denotes the skill corresponding to the exercised question at that time step, and $r_t$ denotes the student’s response result (1 for correct and 0 for incorrect). The vertical axis indicates the skill index. The visualization of knowledge state dynamics generated by DLAKT is shown in Fig. 9.

**Fig. 10:**
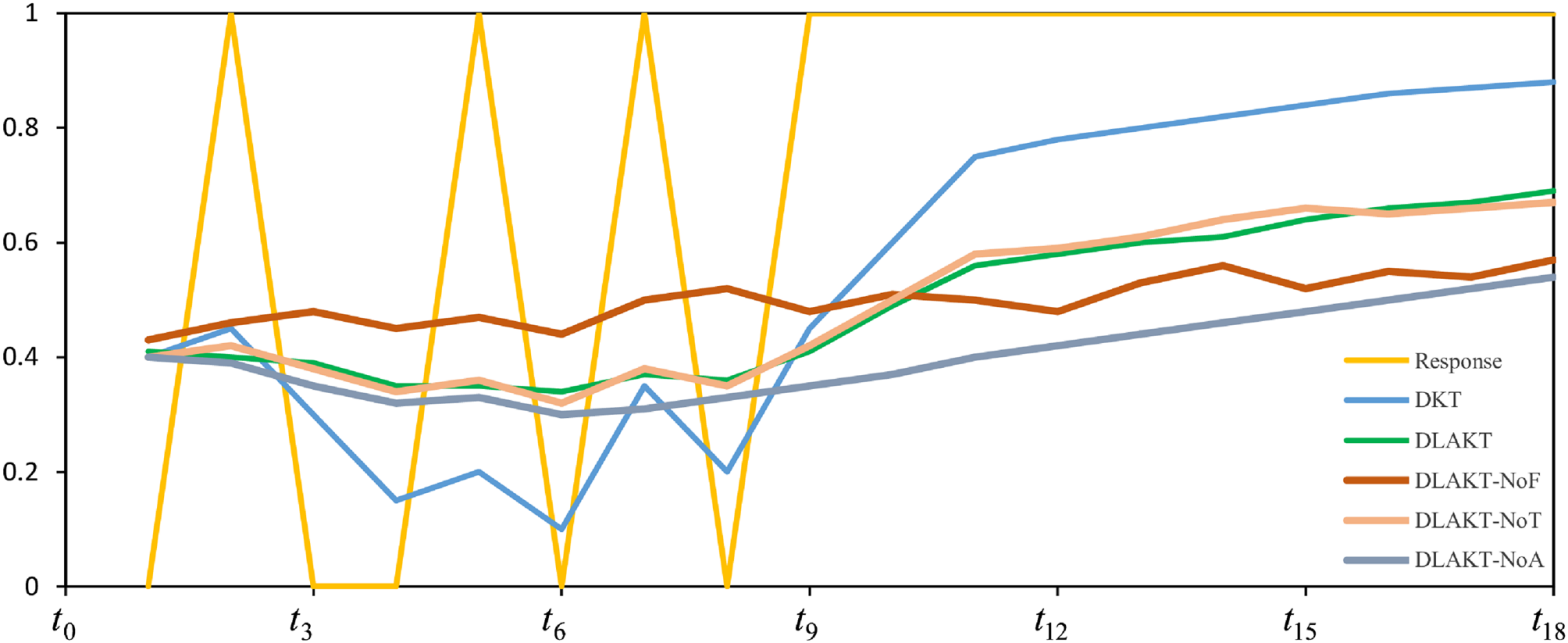
Comparison of knowledge state predictions.

The analysis reveals that for skill $k_2$, as the student continues to practice, the knowledge state predicted by DLAKT shows a steadily increasing trend. This indicates the effectiveness of the proposed model in capturing the dynamics of knowledge accumulation. In contrast, for skill $k_1$, which has not been accessed for an extended period, the predicted knowledge state exhibits a gradual decline, consistent with cognitive forgetting patterns. This demonstrates the ability of the model to reasonably simulate the forgetting mechanism in student learning. Moreover, when the student initially encounters $k_2$ and makes repeated errors, the proposed model maintains a relatively stable knowledge state prediction. This reflects its robustness in resisting noise from occasional incorrect responses and reduces the risk of misjudging mastery based on isolated behaviors. In addition, $k_3$ and $k_4$ exhibit synchronized fluctuations across multiple time steps. Notably, both belong to the *computation ability* dimension in the domain learning ability structure defined in this study. This finding suggests that the proposed model is capable not only of tracking individual skill mastery but also of capturing latent associations between related skills, thereby enhancing the interpretability and adaptability of personalized predictions.

To further validate the advantage of the proposed model in tracing knowledge state stability, we compare DLAKT with other models on the prediction fluctuations over time steps. Focusing on skill $k_2$ (Application: Simple Multiplication), Fig. 10 shows that DLAKT exhibits smoother, more coherent knowledge state transitions compared to DKT, avoiding sharp shifts caused by isolated errors. This highlights the robustness of DLAKT to behavioral noise and the ability of the model to stably capture student learning dynamics. We also visualize three ablated variants (DLAKT-NoA, DLAKT-NoF, and DLAKT-NoT) to examine the role of individual components.

- DLAKT-NoT exhibits minor local fluctuations despite following the overall trend, indicating that the Transformer-based smoothing module plays an auxiliary yet effective role in enhancing temporal continuity.
- DLAKT-NoF tends to overestimate knowledge states in the early stages and underestimate them later, due to its inability to account for knowledge decay, highlighting the necessity of the forgetting mechanism.
- DLAKT-NoA consistently underestimates knowledge acquisition, especially during initial learning phases, revealing the critical role of the accumulation module in capturing progressive learning gains.

Overall, the superior stability of DLAKT results from the joint modeling of knowledge accumulation and forgetting, with Transformer-based smoothing further reducing noise. Compared to DKT and its variants, DLAKT provides more consistent and interpretable knowledge state estimations.

## Conclusion

This paper proposes a Dynamic Domain Learning Ability Enhanced Knowledge Tracing with Stability (DLAKT). The model consists of two core modules. Firstly, the domain learning ability computation module establishes mappings between knowledge points and abilities. It dynamically adjusts the ability improvement rate by integrating student knowledge states, response time, and exercise difficulty, thereby enabling interpretable modeling of students’ multidimensional ability evolution. Secondly, the knowledge state update module introduces three types of behavioral features based on time and frequency, and explicitly models students’ knowledge forgetting and accumulation processes during learning using a memory network. Finally, experimental results demonstrate that DLAKT outperforms existing mainstream models in both prediction accuracy and stability across multiple public educational datasets. The model is particularly effective in long sequence modeling and in capturing individual differences in learning abilities. The approach not only enhances the proposed model capability to continuously represent knowledge states, but also improves the interpretability and robustness of prediction results.

For future work, we plan to explore a bidirectional modeling mechanism between dynamic domain learning ability and knowledge states. In this mechanism, ability feedback influences knowledge state updates, forming a feedback driven system that enhances model expressiveness and personalization.

## Data Availability

We utilized open-source datasets, namely ASSIST12, ASSIST17 and Algebra05, which can be accessed from https://sites.google.com/site/assistmentsdata/home/2012-13-school-data-with-affect, https://sites.google.com/view/assistmentsdatamining/dataset, https://kdd.org/kdd-cup/view/kdd-cup-2010-student-performance-evaluation/Data.
